# Phosphorylation of Mouse Melanopsin by Protein Kinase A

**DOI:** 10.1371/journal.pone.0045387

**Published:** 2012-09-26

**Authors:** Joseph R. Blasic, R. Lane Brown, Phyllis R. Robinson

**Affiliations:** 1 Department of Biological Sciences, University of Maryland, Baltimore County, Baltimore, Maryland, United States of America; 2 Department of Veterinary & Comparative Anatomy, Pharmacology, and Physiology, Washington State University, Pullman, Washington, United States of America; Dalhousie University, Canada

## Abstract

The visual pigment melanopsin is expressed in intrinsically photosensitive retinal ganglion cells (ipRGCs) in the mammalian retina, where it is involved in non-image forming light responses including circadian photoentrainment, pupil constriction, suppression of pineal melatonin synthesis, and direct photic regulation of sleep. It has recently been shown that the melanopsin-based light response in ipRGCs is attenuated by the neurotransmitter dopamine. Here, we use a heterologous expression system to demonstrate that mouse melanopsin can be phosphorylated by protein kinase A, and that phosphorylation can inhibit melanopsin signaling in HEK cells. Site-directed mutagenesis experiments revealed that this inhibitory effect is primarily mediated by phosphorylation of sites T186 and S287 located in the second and third intracellular loops of melanopsin, respectively. Furthermore, we show that this phosphorylation can occur *in vivo* using an *in situ* proximity-dependent ligation assay (PLA). Based on these data, we suggest that the attenuation of the melanopsin-based light response by dopamine is mediated by direct PKA phosphorylation of melanopsin, rather than phosphorylation of a downstream component of the signaling cascade.

## Introduction

The melanopsin photopigment is expressed in intrinsically photosensitive retinal ganglion cells (ipRGCs) in the mammalian retina. This third class of mammalian photoreceptors differs from the classical photoreceptors, the rods and cones, in both function and physiology. The ipRGCs primarily contribute to non-image forming light responses including circadian photoentrainment, pupil constriction, suppression of pineal melatonin synthesis, and direct photic regulation of sleep. The melanopsin-based signaling cascade causes the ipRGCs to depolarize in response to light, in contrast to the light-induced hyperpolarization seen in rod and cone photoreceptors [1]–[4]. Light activation of melanopsin triggers a phototransduction cascade that most likely involves a G_q/11_ based pathway [5]–[7] that leads to activation of TRPC6/7 channels via PLC beta [6], [8]. We have also recently demonstrated that the deactivation of this pathway may involve the phosphorylation of the carboxy tail of light-activated melanopsin by a G-protein coupled receptor dependent kinase (GRK) [9]. This is a common deactivation mechanism observed in vertebrate visual pigments, as well in many other G-protein coupled receptors.

In the work reported here, we demonstrate that melanopsin’s activity can also be modulated by protein kinase A (PKA). PKA is a serine-threonine kinase that is activated by increased levels of cyclic adenine monophosphate (cAMP). In many regions of the nervous system, the neurotransmitter dopamine is known to increase intracellular levels of cAMP via activation of adenylyl cyclase by D1-like dopamine receptors [10]. Dopamine is also an important neurotransmitter in the vertebrate retina, where its levels are regulated both by light and circadian processes [11]. Under constant darkness, dopamine release is controlled by the circadian clock such that dopamine levels are generally higher in the day and lower at night [12]. Under normal lighting conditions, the daytime increase in dopamine is augmented by an additional light-dependent process that increases dopamine levels [13]. Previously, Brown et al demonstrated that signaling from ipRGCs to the suprachiasmatic nucleus (SCN - the home of the master circadian clock) was attenuated during subjective day compared to that recorded during the subjective night [14], but the mechanism of the effect was not reported. Recently, Van Hook et al. have shown that dopamine exposure attenuates the melanopsin-based light response in rat ipRGCs [15], and dopamine levels in the retina are known to vary in a circadian fashion, being highest in the subjective day. The findings reported here help to identify the intracellular targets that mediate dopamine’s effect on melanopsin-based signaling.

One class of cells in the retina that produces and releases dopamine is the dopamanergic amacrine cells, whose dendrites co-stratify with those of one class of melanopsin-expressing RGCs [16]–[19]. It has also been shown that intracellular administration of cAMP to isolated melanopsin-expressing RGCs decreases the amplitude of the melanopsin-based light response [8]. Taken together this suggests a mechanism through which circadian and light-induced fluctuations in retinal dopamine influence melanopsin activity through PKA. The following work demonstrates that PKA phosphorylates mouse melanopsin in a heterologous expression system and this phosphorylation can inhibit melanopsin signaling in HEK cells. It is determined that this inhibitory effect is primarily mediated by sites T186 and S287 located in the second and third intracellular loops, respectively, of melanopsin. Furthermore, it is shown that this phosphorylation can occur *in vivo* using an *in situ* proximity-dependent ligation assay (PLA).

## Results

### Phosphorylation of Melanopsin by PKA

**Figure 1:**
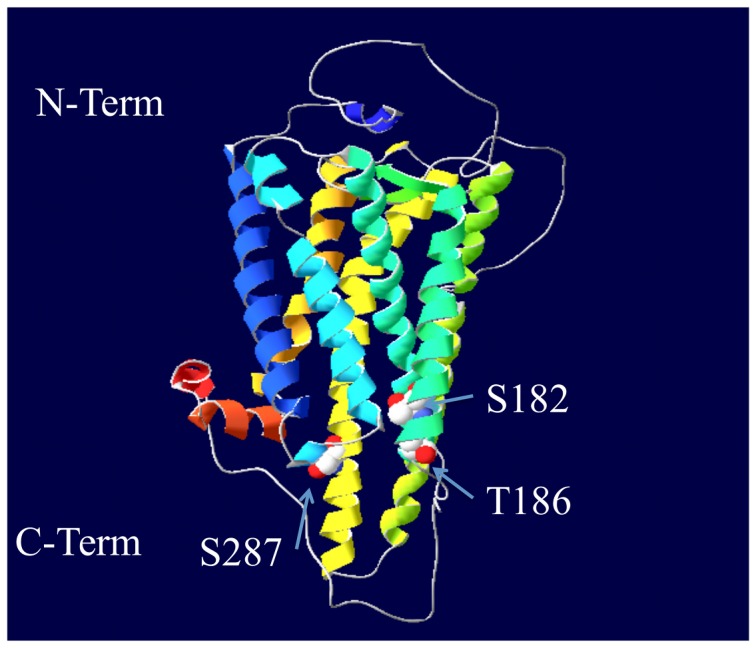
3-dimenstional model of mouse melanopsin highlighting the predicted PKA phosphorylation sites found in intracellular loops. The sites in the C-tail are not depicted. Model constructed by LOMETS [30] modeling server, and sites identified by Group-based Prediction System (GPS 2.0) [20].

**Figure 2:**
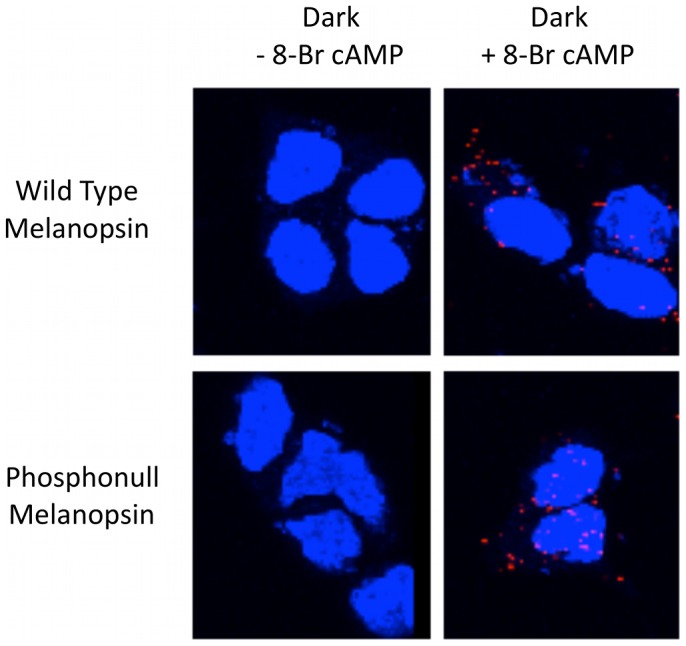
Proximity-dependent ligation assay. Melanopsin-transfected HEK cells were fixed with 4% PFA for 30 min. with or without pretreatment with 200 µM 8-Br cAMP for 30 minutes before fixation. Melanopsin phosphorylation was assayed with the PLA as described in Materials and Methods. The red fluorescence puncta indicates that the antibody bound to melanopsin’s intracellular C-terminal domain is within 40 nm of the phospho-serine antibody when bound to phosphorylation sites in the intracellular loops. Cells visualized by confocal microscopy. Blue staining indicates DAPI staining of cell nuclei. Images represent Z-stacks of images taken through entire cell.

**Figure 3:**
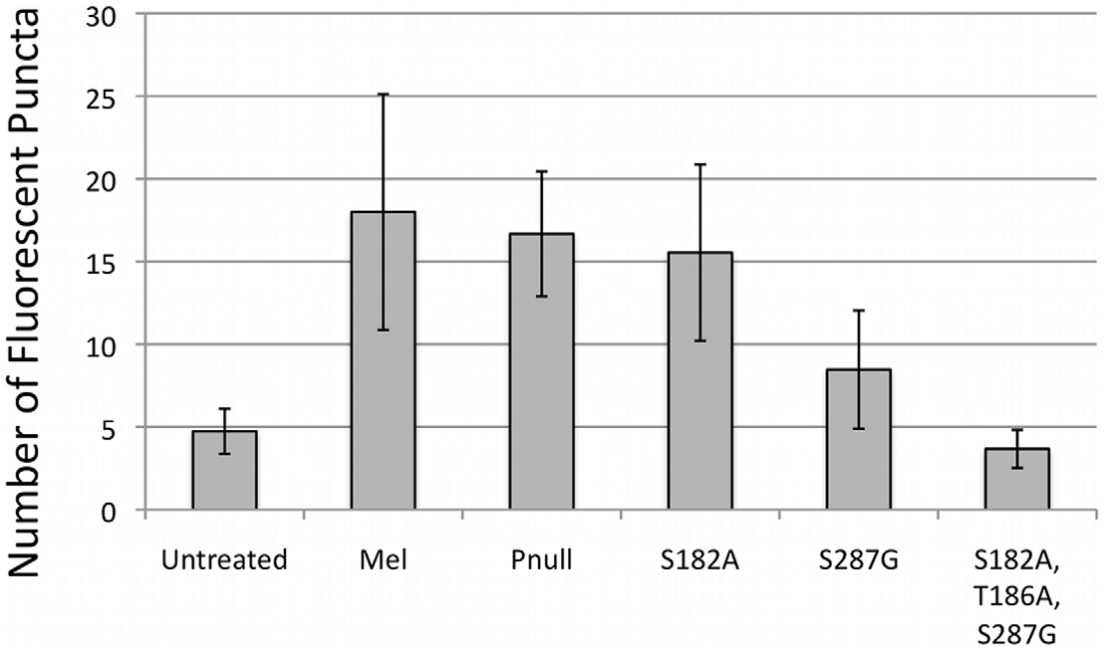
Quantification of the number of fluorescent puncta per cell induced by 8-Br cAMP treatment. HEK cells were transfected with wild type or mutant melanopsin. After 48-hours, cells were treated with 200 µM 8-Br cAMP for 30-minutes, fixed in 4% PFA for 30 minutes, and assayed with the PLA. Cells were imaged by confocal microscopy and the number of fluorescent spots per cell were counted. Expression of melanopsin was confirmed by functional calcium assay. Error bars represent standard deviation of 50 cells counted for each condition pooled from two separate transfections.

Predicted PKA phosphorylation sites within the melanopsin sequence were identified using the Group-based Prediction System (GPS) 2.0 [20]. This method employs a prediction algorithm based on known phosphorylation sites grouped together by kinase family. Using this program at the medium threshold, which has a theoretical false positive rate of 6%, nine predicted PKA were found (Table 1). Three of the predicted sites were contained in the intracellular loops of melanopsin, while the remaining six sites were found in the carboxy tail domain (see Fig.1). The sites in the carboxy tail were not investigated further because PKA dependent modulation was still evident in a melanopsin mutant, in which all potential phosphorylation sites in the C-terminus had been eliminated (see Fig. 2 and 3).

**Table 1:** List of PKA phosphorylation sites predicted by GPS2.0.

Predicted Site	Score
S182	2.151
T186	3.397
S287	2.176
T385	1.965
T389	3.451
S408	3.5
T460	2.53
S480	2.716
S481	2.754

List of the predicted PKA phosphoryation sites in mouse melanopsin. The score is a measure of the similarity of a peptide sequence centered on a phosphorylatable residue to a known phosphorylation site for a given kinase family.

A proximity-dependent ligation assay (PLA) (Olink Biosciences) was used to determine if any of these predicted sites were actually phosphorylated in a cellular environment. The PLA assay is designed to determine the proximity of two epitopes to one another using antibodies specific for each epitope. In this case, antibodies against the carboxy tail of melanopsin and against phosphoserine were used in conjunction with secondary antibodies conjugated with oligonucleotide linkers. If the primary antibodies are bound within ∼ 40 nm of each other, the oligonucleotides will anneal to form a piece of circular DNA, which can then be amplified by a polymerase using rolling circle amplification to produce concatamers of single-stranded copies of the DNA sequence. Addition of fluorescently-labeled oligonucleotide probes specific for the amplified DNA results in a single fluorescent spot for each pair of interacting antibody molecules [21]. Using this method, we have recently shown that the carboxyl terminus of mouse melanopsin is phosphorylated in a light-dependent manner [9]. For these experiments transfected HEK cells were kept in the dark to prevent light-dependent phosphorylation and were treated with the hydrolysis-resistant and membrane-permeant cAMP analog, 8-Br cAMP, to stimulate PKA [22] (Fig. 2). As indicated by the dramatic increase in the number of fluorescent spots, activation of PKA leads to increased phosphorylation, This increase is also seen in cells that express the “phosphonull” melanopsin mutant, which contains no serine or theonine residues in the carboxy tail domain, but retains the putative phosphorylation sites in the intracellular loops (Fig. 2). These results demonstrate that PKA-dependent phosphorylation does not occur on the sites in the carboxy tail.

In order to confirm the importance of these putative PKA phosphorylation sites, each was individually mutated to non-phosphorylatable residues (S182A; T186A; S287G); a double mutant (T186A, S287G) and a triple mutant containing all mutations (S182A, T186A, S287G) were also constructed. These mutant constructs were also examined by PLA in the dark in the presence of 200 µM 8-Br-cAMP (Fig. 3). The quantitation of the fluorescent spots revealed no significant decrease in the number of spots per cell between the wild-type protein and the phosphonull (P-value = 0.50) or between wild type and S182A (P = 0.39), implying that site 182 is not a primary target of PKA phosphorylation. A significant decrease was observed in the number of fluorescent spots between the wild-type protein and S287G (P = 0.0016), and between the wild type and the triple mutant (P = 4.3×10^−6^). The PLA signal remained slightly elevated even in the S287G mutant, and only returned to untreated levels of fluorescence in the triple mutant (S182A, T186A, and S287G). Although S182 may not be the primary target of PKA phosphylation in HEK cells, these results suggest that this residue may still be phosphorylated in the absence of other available sites.

### Phosphorylation of Melanopsin by PKA Reduces Activity

**Figure 4:**
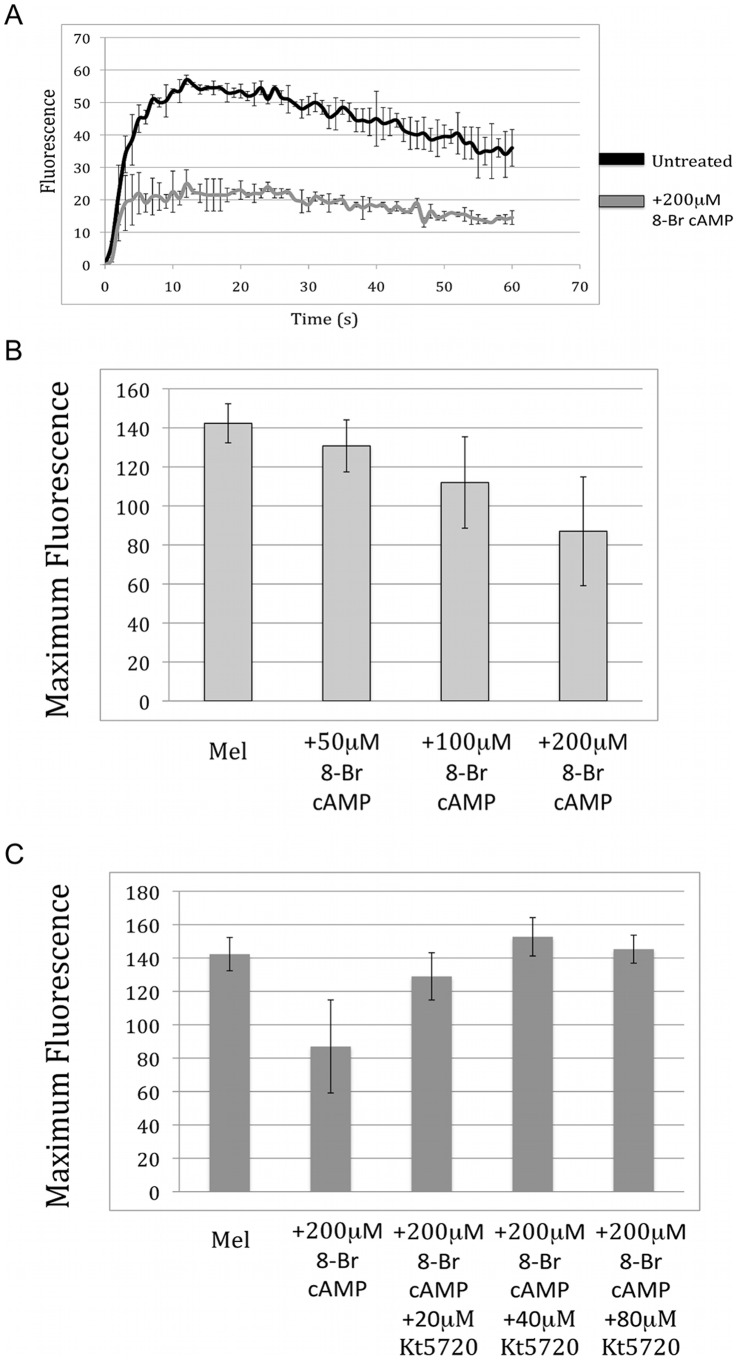
Effect of 8-Br cAMP on light induced calcium mobilization in melanopsin-transfected cells. A) Time course of the calcium response of HEK-293 cells in the presence and absence of 8-Br cAMP. HEK-293 cells were transiently transfected with DNA for wild-type melanopsin. Some cells were treated with 200 µM 8-Br cAMP. Melanopsin signaling was monitored by measuring intracellular calcium levels as described in Methods. B) HEK-293 cells transfected with melanopsin were treated with varying concentrations of 8-Br cAMP to show a concentration dependent decrease in melanopsin signaling. The peak response of the time course is plotted. C) Pre-treatment of transfected cells with the specific PKA inhibitor KT5720 removed the effect of 8-Br cAMP treatment also in a concentration dependent manner. Error bars represent standard deviation.

Having shown that the activation of PKA leads to phosphorylation of melanopsin in the dark, we next examined the effect of this phosphorylation on melanopsin function. This was tested using a kinetic calcium-signaling assay as previously described [9]. It has been shown that melanopsin activation leads to an increase in intracellular calcium in both native ipRGCs and when heterologously expressed in HEK cells [23], [24]. We exploited this fact by monitoring intracellular calcium concentrations using the calcium-sensitive fluorescent dye Fluo-4. This dye has high fluorescence when bound to calcium and low fluorescence in its absence. The absorbance maximum of melanopsin (480 nm) and the excitation maximum of the fluorescent dye (490 nm) are sufficiently close that both can be excited with the same wavelength of light. Therefore, as we monitor fluorescence of Fluo-4, we are also exciting the melanopsin and initiating the phototransduction pathway. In this way, measuring Fluo-4 fluorescence over time can monitor light-dependent activation of melanopsin. The effect of PKA activation on melanopsin signaling was determined by treating melanopsin-transfected cells with 8-Br-cAMP to stimulate PKA before the calcium assay. In these experiments, all cells are from the same melanopsin transfection and express melanopsin at the same level. An example of the calcium response is shown in Fig. 4A. Increasing concentrations of 8-Br cAMP leads to increasing levels of inhibition of the calcium signal (Fig. 4B). Addition of a cAMP analog into the cells could have a variety of effects on the calcium system in the cell [21]. Therefore, to demonstrate that the effect of 8-Br cAMP was caused specifically by PKA, cells were also treated with the PKA inhibitor KT5720 [25]. KT5720 binds to the catalytic subunit of PKA and competitively blocks the binding of ATP. Upon pretreatment of cells with KT5720, the 8-Br cAMP induced inhibition was readily reversed (Fig. 4C).

**Figure 5:**
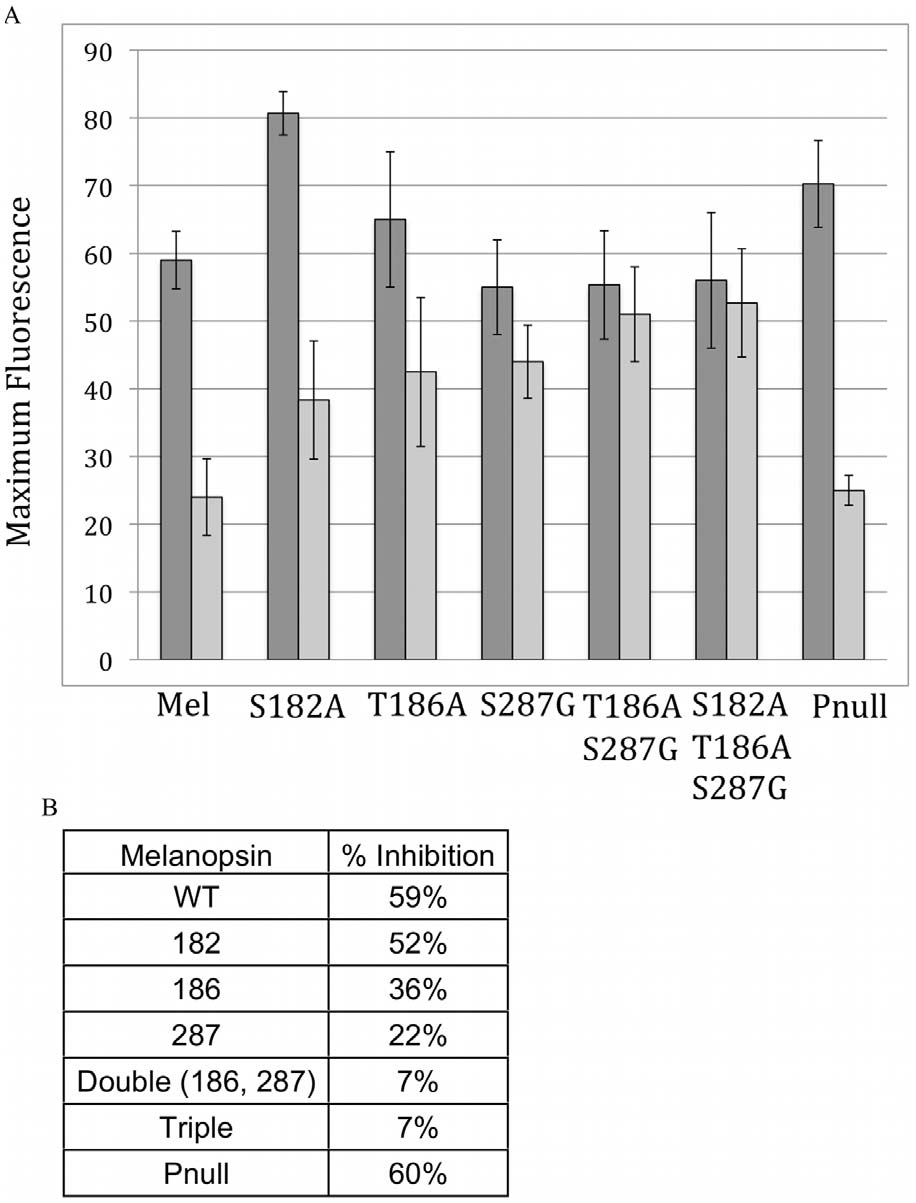
Effect of 8-Br cAMP on mutant and wild type melanopsin calcium signaling. A) Graph of the peak calcium response of melanopsin and melanopsin mutants as measured by fluorescent calcium assay. In black is the average maximum fluorescence for untreated cells, while 200 µM 8-Br cAMP treated response is shown in grey. Error bars represent standard deviation. B) The average percent 8-Br cAMP induced inhibition in signaling is shown.

In order to confirm the importance of the previously identified PKA phosphorylation sites (S182, T186, and S287), mutant melanopsin proteins with were screened using the calcium kinetic assay (Fig. 5A). As predicted from the PLA data, both the phosphonull and S182A mutants showed similar levels of PKA-dependent inhibition as the wild type protein. In contrast, the melanopsin T186A and S287G mutants showed greatly reduced inhibition. Furthermore, as expected, the double and triple knockouts both showed little 8-Br-cAMP dependent inhibition (Fig. 5B).

### Evidence for Phosphorylation of Melanopsin by PKA *in vivo*

**Figure 6:**
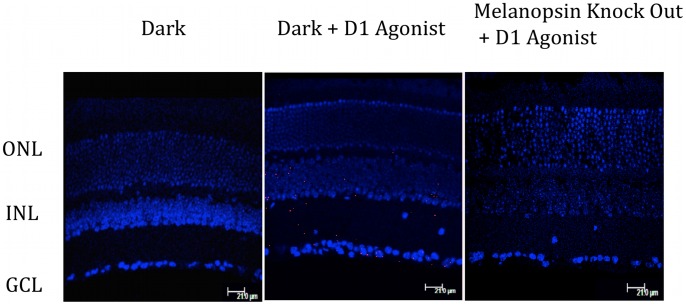
Dopamine agonist induces phosphorylation *in vivo*. PLA was performed on mouse retinal section following treatment with the D1 agonist A68930. Retinal sections (16 mm) were taken from dark-adapted wild type C57/B6 mice (panels labeled Dark, or Dark + D1 agonist) or dark-adapted melanopsin knockout mice (opn4 ^LacZ/LacZ^) (panel labeled Melanopsin knock out + D1 agonisit). Before fixation retina were treated with the dopamine D1 agonist A68930 (labled +D1 agonist) or left untreated (Dark). Outer nuclear layer (ONL): Inner nuclear layer (INL) and Ganglion cell layer (GCL).

To demonstrate if PKA-dependent phosphorylation of mouse melanopsin occurs *in vivo* as it does in transfected cells, we performed a PLA assay using murine retinal sections. We have previously demonstrated light-dependent phosphorylation of the C-terminal tail of melanopsin in the retina, similar to the phosphylation observed in HEK cells, and that it remains largely unphosphorylated in the dark [9]. Dark-adapted retinas were fixed with or without a 30 min prior exposure to 50 nM concentration of the dopamine D1 receptor agonist A68930. An increase in the number of fluorescent spots was seen upon treatment with the D1 agonist as compared to untreated retina in the dark (Fig. 6 A&B). No spots were detected in melanopsin knockout animals treated with D1 agonist (Fig. 6 C).

### Phylogenetic Analysis of Putative Phosphorylation Sites

**Figure 7:**
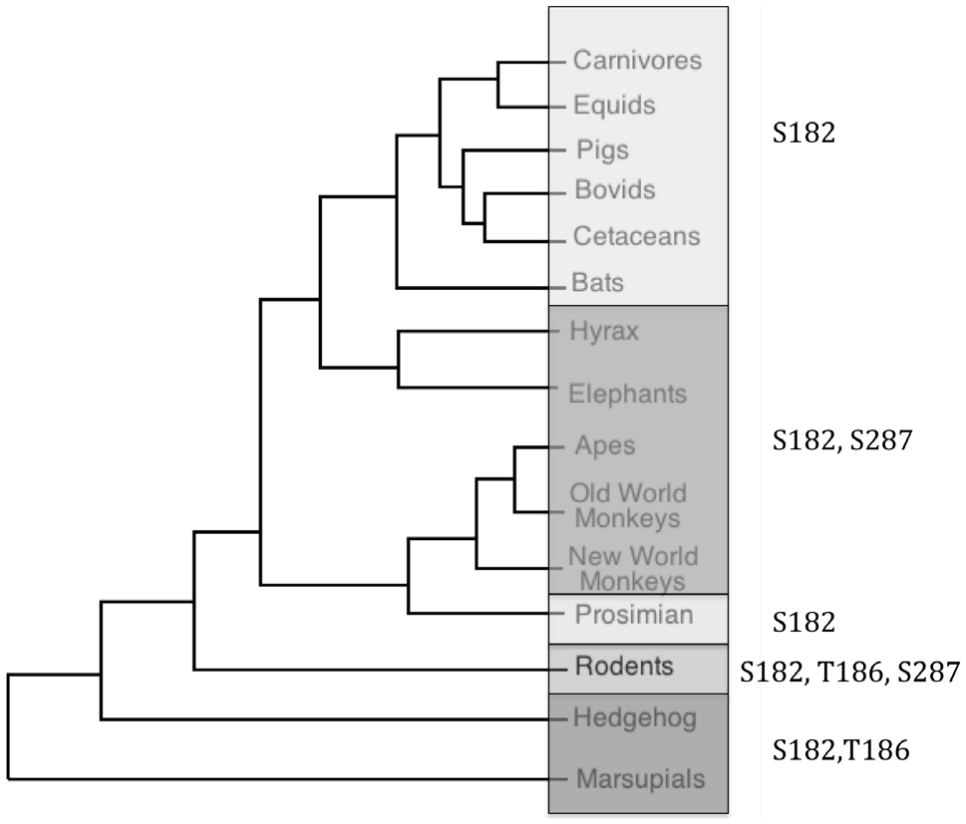
Modified mammalian tree highlighting presence of putative homologous PKA phosphorylation sites. Tree based on (Tree of Life Web Project. 1997. Eutheria. Placental Mammals. Version 01 January 1997 http://tolweb.org/Eutheria/15997/1997.01.01 *in* The Tree of Life Web Project,http://tolweb.org/ *).* Conserved PKA phosphorylation sites are indicated on the right.

**Figure 8:**
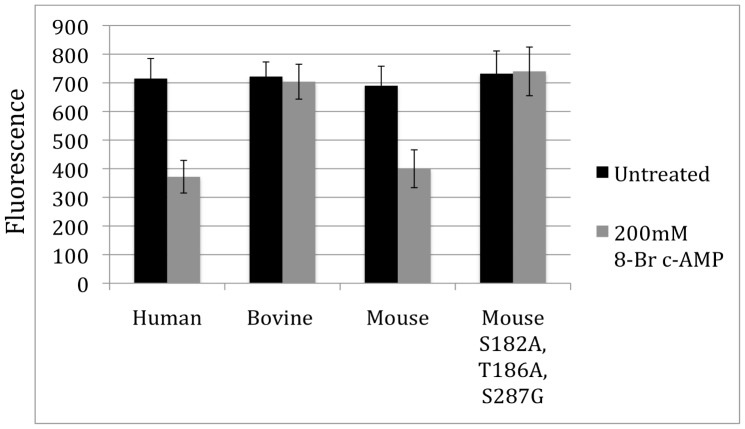
Functional consequences of natural variation in PKA phosphorylation sites. Graph of the peak calcium response of human, bovine, mouse melanopsin and the mouse melanopsin mutant (S182A,T186A, S287G) as measured by the fluorescent calcium assay. In black is the average maximum fluorescence for untreated cells, while 200 µM 8-Br cAMP treated response is shown in grey. Error bars represent standard deviation.

In order to investigate the broader implications of the identified PKA sites on melanopsin, these sites were examined across the twenty-five mammalian melanopsin sequences previously analyzed by Porter and colleagues (Fig. 7) [26]. Sequences were aligned with ClustalW and conservation of the putative PKA sites was examined (see Figure 1 to visualize the sites in a 3 dimensional melanopsin model). Serine 182 is highly conserved across all species. While this site was not found to be functionally relevant in our heterologous assay system, there was still evidence for it being phosphorylated (Fig. 3). In this study, the sites determined to be functional important (T186 and S287) are less well conserved with only the rodents possessing both sites. The marsupials retain only T186, and primates, elephants, and hyrax posses only S287. Interestingly, the monophyletic group including cows, pigs, carnivores, cetaceans and bats possess neither of the two functional sites, implying that fluctuating dopamine levels may not regulate melanopsin signaling in their retinas. To test the effect of PKA activation in these other phylogenetic groups, human melanopsin and bovine melanopsin were also screened in the functional calcium mobilization assay (Fig. 8). As predicted from the mouse mutational analysis, the activity of human melanopsin, which contains both S182 and S287, is inhibited in the presence of 8-Br cAMP, while the activity of bovine melanopsin, which contains none of the functionally relevant residues, is completely insensitive to increasing levels of 8-Br cAMP.

## Discussion

Presented here is the novel finding that melanopsin signaling can be regulated by phosphorylation serine and threonine residues found in intracellular loops two and three by protein kinase A. Phosphorylation occurs at three locations, S182 and T186 of loop two and site S287 of loop three. Of these sites, T186 and S287 appear to be the most functionally important for regulating melanopsin signaling in rodents, and mediate an inhibition of the total amount of calcium mobilized by melanopsin in response to light in transfected HEK cells. These findings identify a potential mechanism for the dopamine modulation of the photoresponse which has recently been reported in rat ipRGCs [15], and they agree with previously published results showing that dialysis of cAMP into ipRGCs during whole-cell recording lead to a 25–30% decrease in the melanopsin-based light response [8]. It has also been shown that dopamine levels in the retina fluctuate throughout the day, peaking just after subjective dawn and remaining high throughout the day. The low point is reached just after onset of subjective night [12]. In agreement with these data, it has recently been shown that there are measurable differences in melanopsin-based signaling to the SCN, one of the major sites of projection of ipRGCs, depending on the time of day. During subjective day, the signal to the SCN was reduced when compared to the signal recorded during the subjective night [14]. It has also been shown that dopaminergic amacrine cells synapse onto melanopsin expressing cells [16], [17]. All of this taken together with the data presented here provides the basis for a model of circadian regulation of melanopsin signaling through dopamine-stimulated PKA phosphorylation. One possible purpose for such a system is for adaptation to constant stimulus throughout the day. In the case of circadian photoentrainment, the most important information to be detected and communicated is the transition from dark to light and from light to dark. Once the onset of light has been detected and communicated to the SCN, the clock has been adjusted at the onset of the signal, and there is no need to continue signaling that the lights are on. The need for attenuation is less obvious for other melanopsin functions, like pupil constriction, which does benefit from continual information about environmental light conditions. However, it is not necessary that all melanopsin functions be regulated in this way. It has been shown that different melanopsin functions may be segregated into different ipRGC cell types [27], and some ipRGC cell types may be more or less sensitive to regulation by PKA. This idea is echoed in the non-mammalian vertebrate sequence alignment showing that one of the melanopsin genes, OPN4x, has retained these sites while the OPN4m has only retained S182.

The conservation of these sites across the 25 species examined here implies that it is advantageous to have at least one of the two sites (T186, S387) found to be functionally important. In the non-mammalian vertebrate species, there is a division between the OPN4m sequences and OPN4x, with OPN4x containing these three phosphorylatable sites, while the OPN4m does not. This may reflect a division of labor between the two genes, with OPN4x being more sensitive to regulation by dopamine than OPN4m. From this current analysis it is unclear if the common ancestor of melanopsin shares these PKA phosphorylation sites. In some species, however, the melanopsin sequence contains none of the identified regulatory phosphorylation sites. Although the third site, S182, is the most conserved residue, its mutation had no effect on the PKA-dependent suppression of melanopsin signaling. Further analysis of this site shows that it is a better match as a protein kinase C (PKC) phosphorylation site, which may provide an explanation for its high degree of conservation.

## Materials and Methods

### Ethics Statement

This study was carried out in strict accordance with the recommendations in the Guide for the Care and Use of Laboratory Animals of the National Institutes of Health. The protocol was approved by the Committee on the Ethics of Animal Experiments of the Washington State University (Permit Number: 3766). To sacrifice animals we anesthetized mice with isofluorane inhalation, followed by cervical dislocation, and all efforts were made to minimize suffering.

### Melanopsin Constructs

Mouse melanopsin construct is as previously described [9]. The human melanopsin construct was kind gift of Dr. Ignacio Provencio (Dept. of Biology, University of Virginia), and was subcloned by PCR methods into the pMT3 vector. Bovine melanopsin was cloned directly from bovine retina using primers designed from the predicted bovine melanopsin sequence (*mouse melanopsin* (GenBank *accession no*. 6693702).

### Kinetic Calcium Imaging

Melanopsin activity was determined using a fluorescent Ca^2+^-imaging assay as previously described [9]. Briefly, HEK-293 cells were transfected with wild type or mutant melanopsin in the pMT3 expression vector using Lipofectamine Plus per the manufacturer’s instructions (Invitrogen). Cells were allowed to grow 24 hrs after transfection, and then released from the plate with 0.25% trypsin (Invitrogen), counted, and re-plated for fluorescent Ca^2+^-imaging at a density of 8×10^4^ cells per well in a 96 well plate with a clear bottom and black walls (Becton-Dickinson). Twenty-four hours after re-plating, cells were washed two times with Hank’s Balanced Salt Solution (HBSS) plus 20 mM HEPES, pH 7.4, and subsequently incubated in HBSS/HEPES, supplemented with 4 µM Fluo-4 AM (Invitrogen), 0.02% pluronic acid (Invitrogen), and 20 µM 11-*cis* retinal. Fluorescent measurements were performed on a Tecan Infinite M200 microplate reader (Tecan Group Ltd.) (EX 485 nm, EM 520 nm), sampling at a rate of 1 Hz for 60 seconds. Background fluorescence was subtracted to account for variation in dye loading and cell density.

### Stimulation and Inhibition of PKA

8-Br-cAMP (Tocris Biosciences) was added to a final concentration of 200 µM (except where noted) 30 minutes prior to reading. To inhibit PKA activity, KT5720 (Tocris Biosciences) was added 10 minutes prior to addition of 8-Br-cAMP.

### Site-directed Mutagenesis

Site directed mutagenesis was performed to remove predicted phosphorylation sites from the intracellular loops of melanopsin. Mutations were introduced by PCR using primers containing one base mismatches corresponding to the desired change in the coding sequence as previously described [28] (Table 2). High fidelity polymerase (Pfx Turbo Invitrogen) was used to minimize unwanted mutations,and desired sequences were confirmed by sequencing.

**Table 2:** List of primers used to create single and double PKA mutants.

Name	Sequence
S182A F	CCACTGGCCACCATCGGCAGGGGAGCCAAAAGACGAACGGCACTCGTCCTGCTAGG
S182A R	CCTAGCAGGACGAGTGCCGTTCGTCTTTTGGCTCCCCTGCCGATGGTGGCCAGTGG
T186A F	GGCCACCATCGGCAGGGGATCCAAAAGGCGAACGGCACTCGTCCTGCTAGGC
T186A R	GCCTAGCAGGACGAGTGCCGTTCGCCTTTTGGATCCCCTGCCGATGGTGGCC
S287G F	GCGGCAGTGGCAGCGGCTGCAGGGTGAGTGGAAGATGGCCAAGGTCGCACTG
S287G R	CAGTGCGACCTTGGCCATCTTCCACTCACCCTGCAGCCGCTGCCACTGCCGC

### Proximity-dependent Ligation Assay

We have previously used this assay to demonstrate that mouse melanopsin’s carboxy tail is phosphorylated in a light-dependent manner both in tissue culture cells and *in vivo* [9]. We use the assay here to probe for PKA-dependent phosphorylation. For experiments with heterologously-expressed melanopsin, HEK-293 cells were transiently transfected with DNA encoding wild-type or mutant melanopsin, seeded onto poly-lysine-coated coverslips 24 hrs post transfection, and grown overnight. Cells were incubated with 20 µM 11-*cis*-retinal for 1 hr, and fixed in ice-cold 4% PFA in the dark, before or after a 30 min incubation in 200 µM 8-Br cAMP. For experiments with endogenous melanopsin, C57/B6 mice were dark adapted overnight (>16 hrs) and sacrificed under dim red light. Animals were enucleated, and eyes were pierced through the cornea with a scalpel to create a large hole to allow solutions access to the retina. Untreated retinas were placed directly into ice-cold 4% PFA for 1-hour, while treated retinas were first soaked in 50 nM of the dopamine D1 receptor agonist A68930 (Tocris Biosciences) for 30 minutes before fixation. Cornea and lens were removed and the remaining eyecup was cryoprotected in 30% sucrose, embedded in OCT, and sectioned at 16 µm on a cryostat. Melanopsin-expressing cells or retinal sections were permeabilized in PBS containing 0.3% Triton-X, and blocked in PBS containing 0.3% Triton-X and 10% normal goat serum. Cells were probed with rabbit anti-melanopsin C-terminal antibody (Thermo Scientific, Cat #PA1-781) and mouse anti-phosphoserine (Sigma Aldrich, Cat# P5747). Specificity of the anti-melanopsin C-terminal antibody has been demonstrated previously [29], and was confirmed by Western blot on wild-type and melanopsin knockout mice. Duolink (Olink Biosciences) *in situ* proximity ligation assay (PLA) was performed according to the manufacturer’s protocol. PLA probes were diluted in 0.1% Triton X-100/PBS/1% fetal calf serum and incubated in a pre-heated humidity chamber for 1 h at 37°C, followed by hybridization, ligation, amplification, and detection. Fluorescence was visualized by confocal microscopy (TCS SP5 Lecia Microsystems). Fluorescent spots were counted and error bars represent standard deviation of 50 cells counted for each condition pooled from two separate transfections.
